# The Mobile Constant, a Self-Reported Method for Shoulder Function Evaluation: Development and Validation Study

**DOI:** 10.2196/63308

**Published:** 2025-09-03

**Authors:** Jingyuan Fan, Tao Zhang, Fanbin Gu, Zhaoyang Wang, Chengfeng Cai, Honggang Wang, Xiaolin Liu, Jiantao Yang, Jian Qi, Qingtang Zhu

**Affiliations:** 1 Department of Microsurgery, Orthopedic Trauma and Hand Surgery The First Affiliated Hospital, Sun Yat-sen University Guangzhou China; 2 Department of Rehabilitation Medicine The First Affiliated Hospital, Sun Yat-sen University Guangzhou China; 3 Department of Hand and Foot Rehabilitation Guangdong Provincial Work Injury Rehabilitation Hospital Guangzhou China; 4 Guangdong Province Engineering Laboratory for Soft Tissue Biofabrication Guangzhou China; 5 Guangdong Provincial Key Laboratory for Orthopedics and Traumatology Guangzhou China

**Keywords:** Constant-Murley Scale, shoulder function evaluation, pose estimation, machine learning, telemonitoring

## Abstract

**Background:**

Shoulder pain is a highly prevalent musculoskeletal disorder that severely compromises patients’ quality of life. The Constant-Murley Scale (CMS) is a well-established method for shoulder function evaluation. However, the necessity of clinician involvement constrains its utility in continuous monitoring. Recent improvements in human pose estimation and inertial sensors provide possibilities for automated functional assessment.

**Objective:**

This study introduces an automated CMS assessment system that can provide objective measuring results using movement images and inertial sensor data (Mobile Constant) and aims to evaluate its reliability by comparison with standard results from human raters.

**Methods:**

The Mobile Constant system integrated subjective symptom questionnaires, range-of-motion analysis, and strength assessment. Patients presenting with shoulder concerns were enrolled consecutively, with movement images and inertial sensor data collected from each participant. The dataset was structured as follows: patients recruited from February to November 2022 at our hospital formed the training set, those enrolled between December 2022 and February 2023 served as the internal validation set, and patients recruited from April to July 2025 at an independent hospital constituted the external validation set. Gold standard assessments were determined independently by 2 raters using standardized protocols. Six machine learning models (logistic regression, k-nearest neighbors, decision tree, support vector machine, random forest, and adaptive boosting) were developed. The reliability of the system was determined by comparison with human raters using differences, Cohen κ coefficients, and intraclass correlation coefficients (ICCs). Agreement across human raters was also evaluated by comparison between 4 independent clinicians.

**Results:**

Data from 141 patients with shoulder pain and stiffness were collected (training set: n=83, 58.9%; internal validation set: n=28; 19.9%; external validation set: n=30, 21.3%). For range-of-motion analysis, the Mobile Constant system showed fair to substantial reliability, achieving κ coefficients ranging from 0.498 to 0.819 and ICCs ranging from 0.898 to 0.956 in the internal validation set. In the external validation set, κ coefficients ranged from 0.198 to 0.699, and ICCs ranged from 0.584 to 0.922. For abduction strength assessment, the k-nearest neighbors model demonstrated substantial reliability, yielding a κ coefficient of 0.707 and an ICC of 0.759 in internal validation and higher agreement in external validation (κ=0.809; ICC=0.906).

**Conclusions:**

The self-reported method for shoulder function evaluation demonstrated substantial agreement with experienced human raters. The proposed system enabled reliable patient-conducted assessment using mobile phone–integrated cameras and inertial sensors and exhibited strong potential for remote monitoring.

## Introduction

### Background

Shoulder pain is an extremely common problem and accounts for approximately 16% of all musculoskeletal disorders [1]. It usually causes substantial pain to patients, impairs work and daily activities, and imposes a heavy societal burden. In the United States, the direct costs of shoulder pain have been estimated at US $7 billion annually [2]. During the treatment and follow-up of patients with shoulder pain, functional assessment is of great importance. The Constant-Murley Scale (CMS) is a well-established method for shoulder function evaluation, first described in 1987 [3] and widely used internationally [4,5]. The CMS, which mainly focuses on the evaluation of shoulder pain and stiffness, consists of 4 subscales: pain, activities of daily living, range of motion (ROM), and strength. The first 2 subscales are subjective items, while the last 2 subscales are objective measurements. The combination of subjective and objective assessments enhances result reliability, but the need for clinician input and the time-consuming procedure limit the use of the CMS in follow-up [6].

To address this problem, several studies have been conducted by modifying the original scale into self-report versions [6,7]. Although these approaches provided usable alternatives and demonstrated acceptable reliability, concerns regarding the subjectivity of results remain [8,9]. Therefore, establishing a self-reported objective measure is key to resolving this issue. With the rapid development of deep learning methods and portable sensors, movement analysis is breaking free from the constraints of measuring equipment. Among various methods, applications based on human pose estimation (HPE) and inertial sensors are the most prevalent.

HPE is a developing technique that can identify the positions of joint landmarks from images or videos, with a number of studies proving its efficiency in remote rehabilitation [10,11] and monitoring [12,13]. Our previous studies have validated the reliability of the HPE algorithm for measuring ROM [14,15]. As ROM assessment in the CMS could be considered as the measurement of multiple joints, the HPE algorithm may also be a potential method for achieving automated scoring. In addition, as a remote motion analysis modality, inertial measurement units (IMUs) have demonstrated clinical utility in gait pattern analysis [16], spasticity quantification [17], and ROM measurement [18] through integration with wearable devices. Park et al [19] suggested the possibility of strength scaling using accelerometer signals; however, the viability and reliability of this method for shoulder strength assessment are still not clear.

### Objectives

In this study, we developed a framework for objective assessment of shoulder function using HPE algorithms and IMU sensors, capable of generating CMS scores through the combined analysis of movement images and kinematic data. The ubiquitous integration of high-definition cameras and IMUs in mobile phones enables patients to collect the required data without dedicated hardware. Accordingly, we named this framework Mobile Constant. The objective of this study was to introduce the system and to evaluate its performance by comparison with human raters.

## Methods

### Participants

Participant enrollment was conducted in 2 phases. Patients from the First Affiliated Hospital of Sun Yat-sen University were recruited from February 2022 to February 2023 for model training and internal validation. Subsequently, a separate cohort of patients was enrolled at Guangdong Provincial People’s Hospital between April and July 2025 to serve as the external validation set. The inclusion criteria were as follows: (1) shoulder pain or reduced mobility, (2) the ability to perform the required functional assessments, and (3) age between 18 and 70 years. The exclusion criteria were as follows: (1) severe upper limb deformities (as these can affect the algorithm’s detection capabilities), (2) pain or reduced mobility in other joints (eg, the elbow joint), and (3) unhealed bone or soft tissue injuries.

### Ethical Considerations

Before the assessment, all participants were informed of the study details and provided written consent. The study protocol was approved by the institutional review board of The First Affiliated Hospital, Sun Yat-sen University (2021-387). To ensure data security and privacy, all image processing and computational analyses were conducted exclusively on local workstations within the institution.

### Data Collection and Parameter Extraction

#### Subjective Parameters

The 2 subjective parameters—pain and activities of daily living—were assessed using Chinese versions of the Constant-Murley questionnaire [20].

#### ROM Measurement

For measuring ROM, patients were asked to complete several movement tasks as defined by the CMS (Multimedia Appendices 1 and 2). Their postures were recorded with mobile phone cameras. Images of external rotation were captured from the anterior side, internal rotation from the posterior side, and elevations from the lateral side. For better presentation, images that did not include the patient’s head or hip were excluded. The eligible images were then collected for model construction.

Landmark detection and parameter extraction were performed using the HPE algorithm BlazePose [21]. The model was run in static image mode, with complexity set to 2, minimum detection confidence set to 0.5, and all other parameters left at default values. As the CMS focuses only on upper limb movement, geometric features were generated from selected landmarks, including the nose, left and right shoulders, left and right elbows, left and right wrists, and left and right hips (Multimedia Appendix 3). To efficiently represent these geometric features, we converted the landmark coordinates into angles between vectors, generating 5112 features using the following algorithm (features based on 2D [x and y] and 3D [x, y, and z] coordinates were extracted separately for comparison):



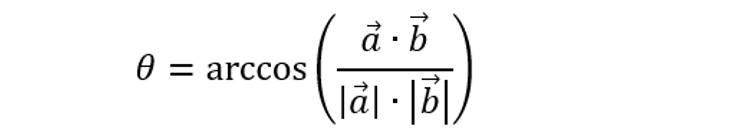



where *a* and *b* are the vectors forming the angle.

#### Strength Measurement

To measure strength, we used the embedded inertial sensors in mobile phones to record shoulder abduction movement data. In this procedure, a mobile phone was attached to the participant’s upper limb. The participant was then asked to repeatedly perform shoulder abduction as quickly and continuously as possible, from a natural resting position to 90° of abduction. Data collection was repeated twice for each patient, with each repetition lasting 10 seconds and sampled at 50 Hz.

The collected data were processed to extract kinematic features representing the movement characteristics of shoulder abduction. The collected sensor data were filtered using a 10 Hz low-pass Butterworth filter implemented with the butter function from the Scipy.signal package in Python (version 3.9; Python Software Foundation). The filter order was set to 8, and the index was 0.3. Next, a 2-second sliding window with a 1-second step size was used to segment the data, resulting in 8 segments per session. From these segments, we extracted a set of time- and frequency-domain parameters (Multimedia Appendix 4) for model construction.

### Data Processing

After data processing, we observed a substantial class imbalance in the data. The proportions of patients with severely impaired shoulder mobility (ROM score <10) and those with completely normal mobility (ROM score=40) were low, at 2.7% (3/111) and 7.2% (8/111) respectively. To address this imbalance and reduce the risk of overfitting, we included data from 5 healthy volunteers as a positive reference and images from patients with severely impaired shoulder function as a negative reference. As the strength measurement data remained imbalanced even after including the volunteer data, we additionally applied oversampling to the minority target variable.

### Data Annotation

#### ROM Annotation

The eligible images were assigned to 2 independent raters for annotation, both of whom had undergone standardized training before scoring. Disagreements were resolved through discussion or consultation with a third investigator until a consensus was reached. These finalized annotations served as the gold standard for the subsequent comparisons. For external rotation, if patients correctly completed the required posture, the images were annotated according to the corresponding item number (classes 1-4). If patients were unable to perform the movement as required, the images were annotated as class 5. For internal rotation, the images were categorized into 6 classes based on the CMS.

#### Strength Measurement

Shoulder strength was measured manually following the procedure described by Constant et al [22]. Measurements were taken at 90° of shoulder abduction in the scapular plane at wrist level using a handheld digital dynamometer (microFET 2; Hoggan Scientific). The final score was defined as the maximum value of 3 repetitions, each separated by a 1-minute interval. Patients who were unable to achieve the test position were assigned a score of 0. The strength values were then converted into 5 annotation classes: 0 (0-5 lb [0-2.3 kg]), 1 (5-10 lb [2.3-4.5 kg]), 2 (10-15 lb [4.5-6.8 kg]), 3 (15-20 lb [6.8-9.1 kg]), and 4 (>20 lb [>9.1 kg]).

### Model Construction

The collected data, including images of external and internal rotation as well as sensor data from shoulder abduction, were used for model construction. Machine learning models corresponding to these 3 items of the CMS (external rotation, internal rotation, and strength) were built to perform automatic rating.

Before training, principal component analysis was applied to reduce the dimensionality of the features. Six machine learning models—logistic regression, k-nearest neighbors (KNN), decision tree, support vector machine, random forest, and adaptive boosting—were then constructed for each item. Grid search [23] was used to identify the optimal hyperparameters and classifier structures. The classifiers were trained on the training set, and internal validation was performed using 5-fold cross-validation.

### Results Processing

Model predictions were converted into scores for interpretation and comparison. For external rotation, the score was determined by whether the prediction output matched the corresponding item number: a match was judged as correct and a mismatch as incorrect. For internal rotation, scores were assigned directly according to the model outputs. For strength, the final score was determined using a voting mechanism, with the most frequently predicted class taken as the final result. Patients who were unable to achieve 90° of shoulder abduction were assigned a score of 0. The flowchart illustrating this process is presented in Multimedia Appendix 5.

### Model Evaluation

The performance of the classifiers was first evaluated by assessing classification accuracy for each item and the total score. To assess the effect of the data processing techniques, three sets of comparisons were performed: (1) 2D features versus 3D features, (2) inclusion versus exclusion of positive and negative reference data, and (3) use versus nonuse of oversampling. Confusion matrices were used to illustrate discrepancies between classifier predictions and the gold standard. Next, Cohen κ coefficients and intraclass correlation coefficients (ICCs) were used to assess the reliability. The results of the κ coefficient were interpreted using the widely accepted benchmark proposed by Landis and Koch [24]: poor agreement (κ≤0), slight agreement (0<κ≤0.20), fair agreement (0.20<κ≤0.40), moderate agreement (0.40<κ≤0.60), substantial agreement (0.60<κ≤0.80), and almost perfect agreement (κ>0.80). ICC values were calculated using a 1-way random-effects model with single-rater design and interpreted as follows: unacceptable (<0.20), questionable (0.20-0.40), good (0.41-0.60), very good (0.61-0.80), and excellent (0.81-1.00).

To further evaluate the performance of the proposed method, 4 trained human raters were recruited to independently perform ROM scoring. The results of the proposed method were then compared with the gold standard. After model construction for the 3 items, the total CMS score was calculated and compared with the gold standard. Differences were reported as mean (SD).

### External Validation

After model evaluation, the optimal model and data processing technique, as determined on the internal validation set, were applied to the external validation set. The reliability of the Mobile Constant system was subsequently validated against manual evaluations performed by physicians. Agreement between system outputs and expert assessments was quantified using both Cohen κ coefficients and ICCs. Furthermore, confusion matrices were used to visualize discrepancies between system predictions and physician evaluations.

### Experimental Environment

Models were developed and trained using *Scikit-learn* in Python 3.9 on a Windows 11 system with an Intel i7-11800H central processing unit. Random oversampling was performed with *Imbalanced-learn*. Feature extraction was conducted using *MediaPipe* for images and the *Signal* library in Python for inertial signals. Additional packages included *NumPy*, *Pandas*, *OS*, and *Matplotlib*. The κ coefficient was calculated with the kappa2 function from the *Irr* package in R (version 4.3.3; R Foundation for Statistical Computing).

## Results

### Clinical Characteristics

**Figure 1:**
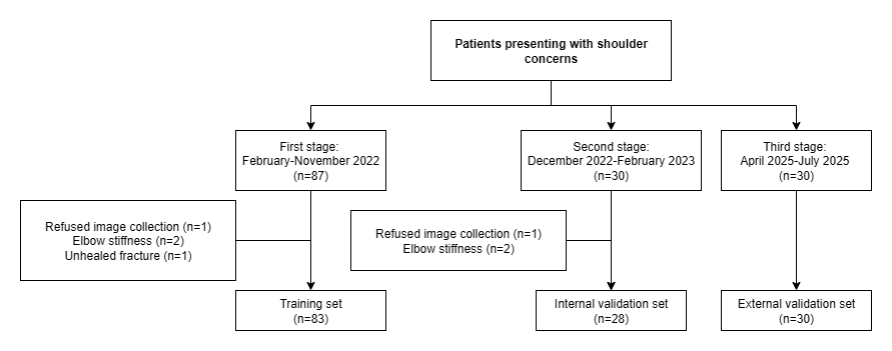
Flowchart of patient inclusion.

During data collection, 117 patients presenting with shoulder concerns were recruited at our hospital. Of these 117 patients, 6 (5.1%) were excluded for the following reasons: refusal of image collection (n=2, 33%), elbow stiffness (n=3, 50%), and unhealed fracture (n=1, 17%). Consequently, 111 patients formed the dataset for model training and internal validation, including 68 (61.3%) male individuals and 43 (38.7%) female individuals, with a mean age of 50.3 (range 17-91, SD:18.0) years. For external validation, 30 consecutive patients presenting with shoulder concerns were enrolled, including 12 (40%) male individuals and 18 (60%) female individuals, with a mean age of 43.4 (range 19-77, SD:16.9) years. The flowchart of patient inclusion is shown in Figure 1, and the clinical characteristics of the patients are presented in Table 1.

**Table 1:** Patient characteristics (N=141).

Characteristics		Training set (n=83), n (%)	Internal validation set (n=28), n (%)	External validation set (n=30), n (%)
**Sex**				
	Male	30 (36)	13 (46)	12 (40)
	Female	53 (64)	15 (54)	18 (60)
**Age (years)**				
	<30	9 (11)	3 (11)	9 (30)
	30-40	10 (12)	6 (21)	5 (17)
	40-50	21 (25)	5 (18)	4 (13)
	50-60	23 (28)	7 (25)	8 (27)
	>60	20 (24)	7 (25)	4 (13)
**Affected side**				
	Left	38 (46)	15 (54)	14 (47)
	Right	45 (54)	13 (46)	16 (53)
**Diagnosis**				
	Scapulohumeral periarthritis	25 (30)	8 (29)	7 (23)
	Rotator cuff injury	20 (24)	9 (32)	15 (50)
	Fracture-dislocation	13 (16)	6 (21)	3 (10)
	Shoulder stiffness	11 (13)	1 (4)	1 (3)
	Shoulder impingement	10 (12)	3 (11)	2 (7)
	Tumor	2 (2)	0 (0)	1 (3)
	Muscle strain	2 (2)	1 (4)	1 (3)

### Reliability of Machine Learning Classifiers

#### ROM Scoring Performance

With minimum detection confidence set to 0.5, the HPE algorithm successfully extracted pose landmarks from 95.7% (850/888) of the included images. The detection rates were 96.1% (533/555), 99.1% (110/111), 87.4% (97/111), and 99.1% (110/111) for external rotation, internal rotation, forward elevation, and lateral elevation, respectively.

To optimize model performance, several data processing methods were conducted, including (1) the use of 3D parameters, (2) the incorporation of positive and negative reference data during training, and (3) class-balance training through oversampling. The results indicated that the highest accuracy was achieved in the model using 2D parameters with positive reference data (Tables 2 and 3). For external rotation, the support vector machine achieved the highest accuracy (92/112, 82.1%). For internal rotation, the best accuracy was obtained by both the random forest and KNN (23/28, 82%).

**Figure 2:**
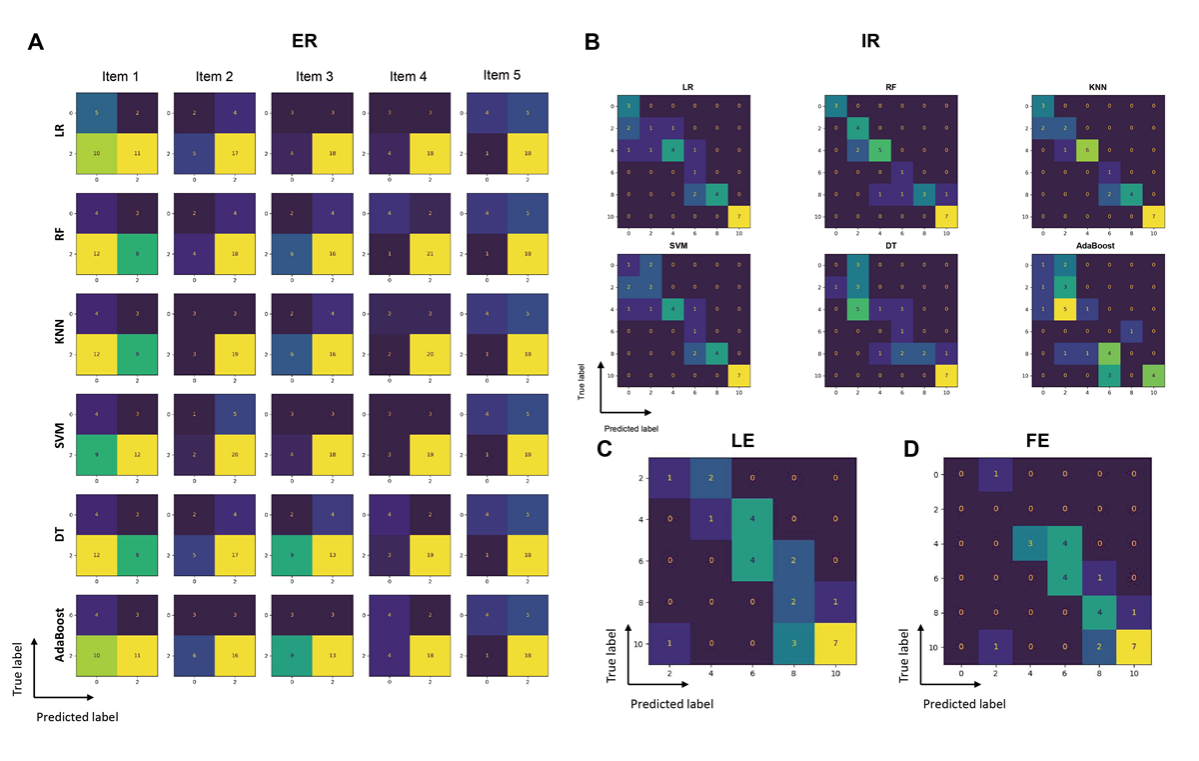
Confusion matrices of classification outcomes for range-of-motion tasks. AdaBoost: adaptive boosting; DT: decision tree; KNN: k-nearest neighbors; LR: logistic regression; RF: random forest; SVM: support vector machine.

Next, the classification results were converted into CMS scores for interpretation (Multimedia Appendix 5). Confusion matrices summarizing true positives, true negatives, false positives, and false negatives are presented in Figure 2.

The reliability of the proposed method was further assessed by comparison with human raters. As shown in Table 4, the κ coefficients for the Mobile Constant method ranged from 0.389 to 0.819 (*P*<.01) for ROM measurements, and the ICCs ranged from 0.898 to 0.956. These values indicate fair to almost perfect agreement with the gold standard. The highest consistency was observed for internal rotation, while lateral elevation showed the lowest agreement. Notably, the proposed method achieved performance comparable to that of trained human raters.

**Table 2:** Summary of the effect of data processing techniques on external rotation.

NR^a^ data	PR^b^ data	2D or 3D	Model accuracy (%)					
			LR^c^	RF^d^	KNN^e^	SVM^f^	DT^g^	AdaBoost^h^
−^i^	−	2D	70.5	65.2	68.8	72.3	47.3	58.9
+^j^	−	2D	79.5	73.2	76.8	75.9	71.4	67.9
−	+	2D	69.6	67	65.2	72.3	54.5	61.6
+	+	2D	76.8	76.8	76.8	82.1	71.4	69.6
+	+	3D	72.5	80	79.2	80	73.3	73.3

^a^NR: negative reference (patients with severe impairment).

^b^PR: positive reference (healthy volunteers).

^c^LR: logistic regression.

^d^RF: random forest.

^e^KNN: k-nearest neighbors.

^f^SVM: support vector machine.

^g^DT: decision tree.

^h^AdaBoost: adaptive boosting.

^i^Excluded.

^j^Included.

**Table 3:** Summary of the effect of data processing techniques on internal rotation^a^.

PR data	2D or 3D	Model accuracy (%)					
		LR^b^	RF^c^	KNN^d^	SVM^e^	DT^f^	AdaBoost^g^
−^h^	2D	53.6	50	42.9	53.6	42.9	32.1
+^i^	2D	71.4	82.1	82.1	67.9	50	32.1
+	3D	60	66.7	76.7	73.3	56.7	50

^a^Only positive reference (PR) data (healthy volunteers) were used.

^b^LR: logistic regression.

^c^RF: random forest.

^d^KNN: k-nearest neighbors.

^e^SVM: support vector machine.

^f^DT: decision tree.

^g^AdaBoost: adaptive boosting.

^h^Excluded.

^i^Included.

**Table 4:** Summary of the κ coefficients and intraclass correlation coefficients (ICCs) between human raters and the Mobile Constant method.

Variables	External rotation			Internal rotation			Lateral elevation			Forward elevation	
	κ coefficient	ICC	κ coefficient		ICC	κ coefficient		ICC	κ coefficient		ICC
Mobile Constant	0.548	0.899	0.819		0.956	0.389		0.927	0.498		0.898
Physician A	0.954	0.987	0.880		0.980	0.748		0.952	0.962		0.987
Physician B	0.584	0.862	0.719		0.928	0.328		0.788	0.822		0.939
Physician C	0.324	0.670	0.695		0.892	0.520		0.880	0.460		0.791
Physician D	0.816	0.974	0.728		0.930	0.486		0.879	0.860		0.970

#### Strength Scoring Performance

At the model training stage, 83 patients and 5 healthy volunteers completed the data collection procedure, generating 176 records. After excluding ineligible data, 163 (92.6%) of the 176 records remained for model construction. At the internal validation stage, 56 records from 28 patients were collected for model evaluation.

As shown in Multimedia Appendix 6, the KNN model achieved the highest accuracy, followed by random forest, adaptive boosting, support vector machine, decision tree, and logistic regression. The oversampling method had a negligible effect on classification accuracy. However, we validated the impact through confusion matrices, which showed that models without oversampling performed poorly in classifying minority class samples (Multimedia Appendix 7). Therefore, the oversampling method was ultimately adopted for subsequent analyses.

**Figure 3:**
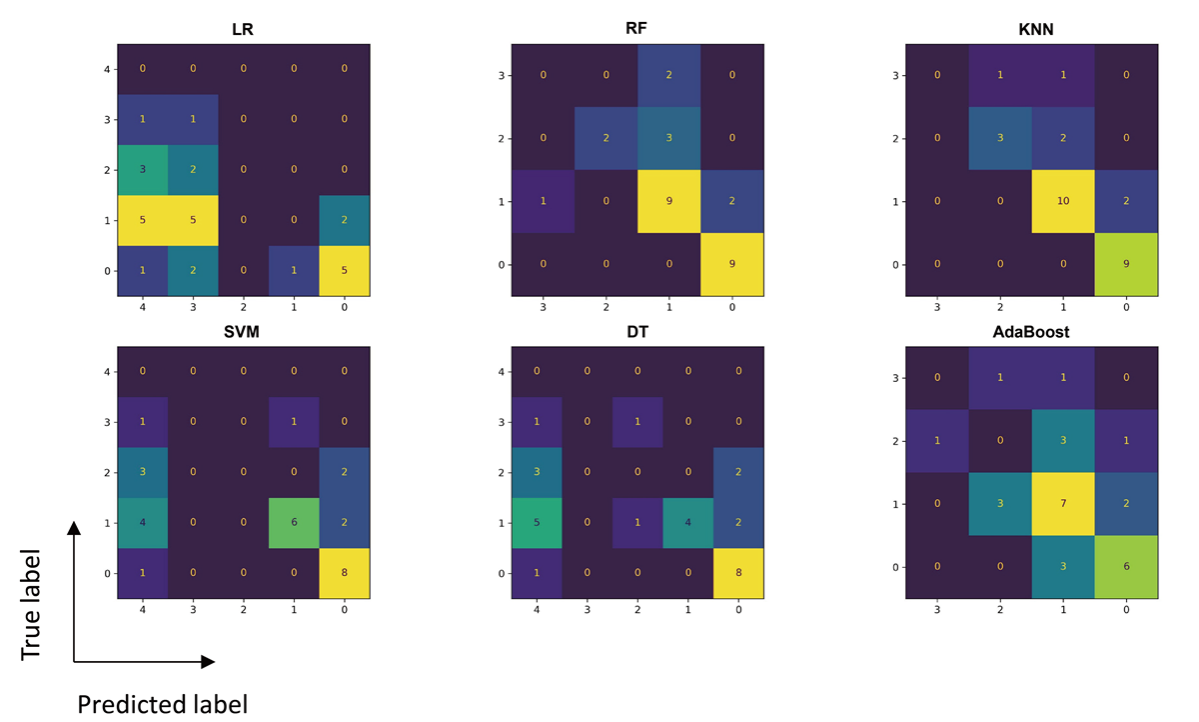
Confusion matrices of machine learning outcomes for strength tasks. AdaBoost: adaptive boosting; DT: decision tree; KNN: k-nearest neighbors; LR: logistic regression; RF: random forest; SVM: support vector machine.

Next, the classification results were converted into the strength scores. The results of the classifiers were first combined using majority voting, and larger value of repetitions was identified as the final result. As shown in Figure 3, the best performance was achieved by the KNN model, with an accuracy of 78.6% (22/28).

In addition, the reliability of the proposed method was evaluated against gold standard methods. In this comparison, the KNN model demonstrated the highest reliability with a κ coefficient of 0.707 and an ICC of 0.759 (Table 5).

The scoring results for patients in the internal validation set, assessed by the Mobile Constant and the gold standard, are summarized in Table 6. The mean difference between the 2 methods was 0.51 (SD 4.51).

**Table 5:** Summary of the κ coefficients and intraclass correlation coefficients (ICCs) for strength assessment.

Model	κ coefficient	*P* value	ICC	*P* value
LR^a^	0.185	.01	0.089	.32
RF^b^	0.547	<.001	0.567	.001
KNN^c^	0.707	<.001	0.759	<.001
SVM^d^	0.287	.003	0.305	.05
DT^e^	0.274	.003	0.293	.06
AdaBoost^f^	0.308	.02	0.474	.004

^a^LR: logistic regression.

^b^RF: random forest.

^c^KNN: k-nearest neighbors.

^d^SVM: support vector machine.

^e^DT: decision tree.

^f^AdaBoost: adaptive boosting.

**Table 6:** Summary of the differences between Mobile Constant and the gold standard.

Variables			Patients, n		Mobile Constant, mean (SD)		Gold standard, mean (SD)		Difference, mean (SD)
**ROM^a^**									
	ER^b^	28		7.86 (3.44)		7.57 (3.71)		0.29 (1.61)	
	IR^c^	28		5.43 (3.65)		5.71 (3.52)		0.29 (1.05)	
	LE^d^	28		7.14 (2.69)		7.00 (2.91)		0.37 (1.11)	
	FE^e^	27		7.33 (2.29)		6.96 (2.79)		0.14 (1.08)	
Strength			28		3.75 (3.50)		5.00 (4.51)		1.25 (2.59)

^a^ROM: range of motion.

^b^ER: external rotation.

^c^IR: internal rotation.

^d^LE: lateral elevation.

^e^FE: forward elevation.

#### External Validation

On the basis of the performance in the internal validation set, models trained with 2D parameters and both negative and positive reference data were used. We selected the support vector machine model for external rotation assessment, the random forest model for internal rotation scoring, and the KNN model with oversampling for strength evaluation. The BlazePose algorithm achieved landmark detection rates of 97% (29/30 patients) for lateral elevation, 90% (27/30 patients) for forward elevation, 98.7% (148/150 frames across all patients) for external rotation, and 93% (28/30 patients) for internal rotation. Subsequent comparisons with manual assessments revealed substantial agreement across all tasks. Consistent with the observations in the internal validation set, external rotation exhibited the lowest interrater agreement (κ=0.198; ICC=0.584), while internal rotation showed the highest (κ=0.699; ICC=0.922). For strength assessment, the Mobile Constant system achieved significant agreement with manual ratings (κ=0.809; ICC=0.906). Agreements between model predictions and manual assessments are presented in confusion matrices in Multimedia Appendix 8. The overall mean difference between manual evaluations and system results was 1.21 (SD 3.66; Table 7).

**Table 7:** Summary of the reliability of Mobile Constant in the external validation set.

Variables	Patients, n	κ coefficient	*P* value	ICC	*P* value	Difference, mean (SD)
ER^a^	29	0.198	.046	0.584	<.001	1.31 (2.29)
IR^b^	28	0.699	<.001	0.922	<.001	0.57 (1.20)
LE^c^	29	0.614	<.001	0.789	<.001	−0.21 (1.45)
FE^d^	27	0.570	<.001	0.875	<.001	−0.22 (1.01)
Strength	30	0.809	<.001	0.906	<.001	0.10 (0.48)

^a^ER: external rotation.

^b^IR: internal rotation.

^c^LE: lateral elevation.

^d^FE: forward elevation.

## Discussion

### Principal Findings

#### Overview

Our study introduced the Mobile Constant method and demonstrated that it was an objective, valid, and reliable instrument for assessing patients with shoulder concerns. This technique makes it possible for patients to perform assessments of shoulder function using only a mobile phone. Mobile Constant could be used as an objective tool in clinical work, especially for remote monitoring.

#### ROM Findings

The original CMS consists of 4 subscales, including 2 subjective items and 2 objective measurements. In our method, the 2 subjective parameters were evaluated using Chinese versions of patient-reported questionnaires [20]. ROM was then assessed using the HPE-based method, which can detect human landmark coordinates and provide quantitative analysis of movements. Although the reliability of the HPE algorithm for measuring ROM has been validated [14,15], methods for conducting movement scoring using this approach remain unclear. The primary challenge stems from the subjective assignment of scores and the absence of quantitative criteria. In this study, we demonstrated that machine learning classifiers using kinematic parameters represent a reliable solution. Original scoring was converted into movement classifications, which demonstrated good reliability.

Although this performance is still far from perfect, it is still comparable to the interrater consistency reported in our study and previous research. Rocourt et al [25] reported substantial variability in interrater reliability for ROM in CMS assessment, with ICCs of 0.460 for external rotation and 0.854 for internal rotation. Blonna et al [26] further identified that physician expertise can impact scoring accuracy, with limits of agreement reaching 7.3 for external rotation and 3.7 for internal rotation.

In our study, the Mobile Constant achieved ICCs of 0.899 for external rotation and 0.956 for internal rotation, with a mean difference of <1, indicating that the proposed method demonstrated reliability comparable to that of human raters.

In addition, our results showed performance comparable, if not superior, to previous research on remote evaluation based on the CMS. Levy et al [6] designed a patient-based questionnaire, reporting consistency with the human rater ranging from 0.80 to 0.90. Similar findings were observed for the Auto-Constant questionnaire [7], which reported an ICC of 0.85 for ROM. These results suggest that our HPE-based method provides acceptable reliability and holds potential as a useful tool for the remote assessment of shoulder movements.

#### Strength Findings

The remote assessment of shoulder abduction strength presents several challenges. First, patients may have difficulty understanding and adhering to the testing protocol in unsupervised settings. In addition, the standardized measuring instrument is typically unavailable outside clinical environments. Recent studies have developed simplified frameworks for remote strength assessment. The most commonly used method is to ask patients whether they can lift objects (eg, a water bottle, packages containing flour or sugar, or a container of milk) while performing abduction [6,7]. This approach is feasible for remote measurement and has demonstrated good reliability, with an ICC of 0.57 [7]. However, results could be impacted by subjectivity and variations in object weight. In this study, we used embedded IMU sensors to extract kinematic data “and train machine learning classifiers. Our method achieved an accuracy of 79% (22/28) in the internal validation set and 73% (22/30) in the external validation set, demonstrating an objective approach to evaluating shoulder strength with good reliability.

#### Effect of Data Processing Approaches

In addition, we conducted tests to evaluate the effect of different data processing approaches. First, we compared the accuracy of models constructed using 2D versus 3D coordinates. Although 3D coordinates provide more detailed pose information and thus may help identify joint positions, which is essential for distinguishing similar postures, the results surprisingly indicated that incorporating 3D information deteriorated classifier performance. We attribute this mainly to estimation errors in 3D pose estimation, which remains a challenging problem in computer vision [21]. These findings also suggest that with the evolution of 3D pose estimation methods, the performance of HPE-based functional assessment would further improve. The next comparison tested the effect of including reference data. Positive reference data, collected from healthy volunteers, were used to demonstrate standard movements to the models, while negative reference data from patients with severely impaired shoulder function were used to present compensatory movements. Interestingly, we found that using negative reference data or combining positive and negative reference data improved model accuracy. Our results suggest a possible method to improve model performance when patient data are insufficient for model training. We also evaluated the effect of oversampling, which addressed the uneven distribution of strength data. Although overall classification accuracy seemed to decline after oversampling, the classifiers exhibited better performance in distinguishing instances in minority groups, as shown in the confusion matrices. This improvement indicates that classifiers possess practical ability in clinical settings because they can detect less common cases rather than simply grouping all samples in the majority class.

### Limitations

Our study has several limitations. The primary limitation is the relatively small sample size, which had direct methodological implications beyond the risk of overfitting. Initially, we used a 10-fold cross-validation strategy, but the small size of the resulting validation splits led to significant instability in performance metrics across folds. This confirmed that for a dataset of our size and distribution, 10-fold cross-validation was not a reliable method for model evaluation. To address this instability, we adopted a 5-fold cross-validation approach. By creating larger validation sets (20% vs 10% of the data), this method produced more stable and trustworthy performance assessments, representing a necessary trade-off to ensure the reliability of our findings. In addition, images in this study were collected in hospitals, the observer was familiar with the procedure, and all patients presented good compliance. If a patient cannot correctly follow instructions or cooperate, model performance would require further validation. In future applications, a real-time feedback module should be incorporated to assess image quality and minimize performance degradation caused by insufficient compliance. Moreover, we recognized that precise localization of key points by HPE algorithms is critical for accurate predictions. In future work, we will implement more advanced algorithms to further improve system performance.

### Conclusions

This study successfully established the Mobile Constant scoring method by combining questionnaire, image, and IMU data and demonstrated its feasibility. Moreover, its performance was validated in patients with shoulder concerns, and the method showed good reliability compared to human observers. This work also presented a method of applying an HPE algorithm to assess shoulder function. The proposed method enables patients to obtain an objective evaluation of shoulder function using only a mobile phone.

## Appendix

Multimedia Appendix 1 Example of movements in Constant-Murley scoring method.

Multimedia Appendix 2 Structure of the Mobile Constant scoring method.

Multimedia Appendix 3 Demonstration of the selected landmarks (marked with red).

Multimedia Appendix 4 Flowchart of the model inference.

Multimedia Appendix 5 Comparison of the performance of classifiers with or without oversampling techniques. A) without oversampling; B) with oversampling. (Note: Patients with shoulder abduction angles ≤90° were excluded. Data from 19 patients are shown, each with two repeated measurements.

Multimedia Appendix 6 Summary of the performance of classifiers in the external validation set.

Multimedia Appendix 7 The calculation of the kinemetric parameters.

Multimedia Appendix 8 Comparison of the accuracy between classifiers with or without the oversampling method.

## Abbreviations

CMS: Constant-Murley Scale
HPE: human pose estimation
ICC: intraclass correlation coefficient
IMU: inertial measurement unit
KNN: k-nearest neighbors
ROM: range of motion

## Data Availability

The coordinate data that support the findings of this study are available from the corresponding author on reasonable request. The original images cannot be shared due to ethical restrictions.
