# Supplementary figures and images for: The Mobile Constant, a Self-Reported Method for Shoulder Function Evaluation: Development and Validation Study
Source: J Med Internet Res. 2025 Sep 3;27:e63308. doi: 10.2196/63308 (PMC12444215; doi:10.2196/63308)

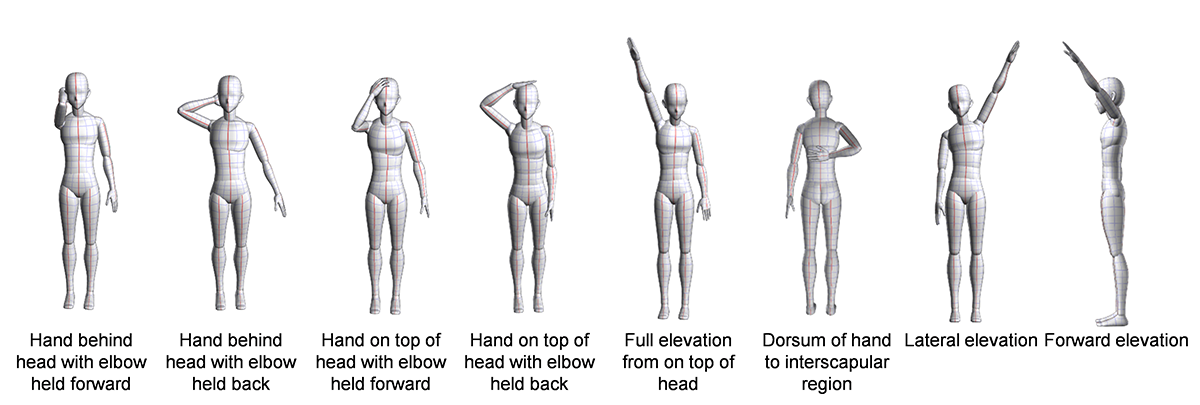

Supplement: Multimedia Appendix 1 [file jmir_v27i1e63308_app1.png]

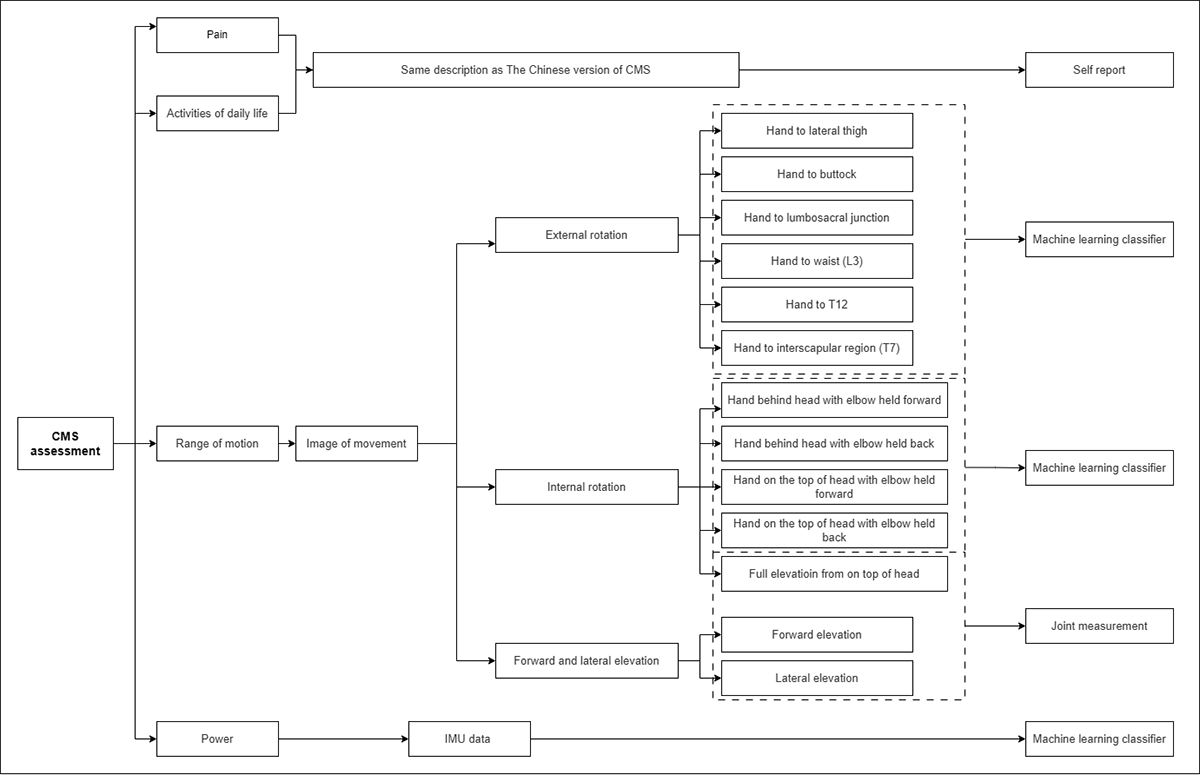

Supplement: Multimedia Appendix 2 [file jmir_v27i1e63308_app2.png]

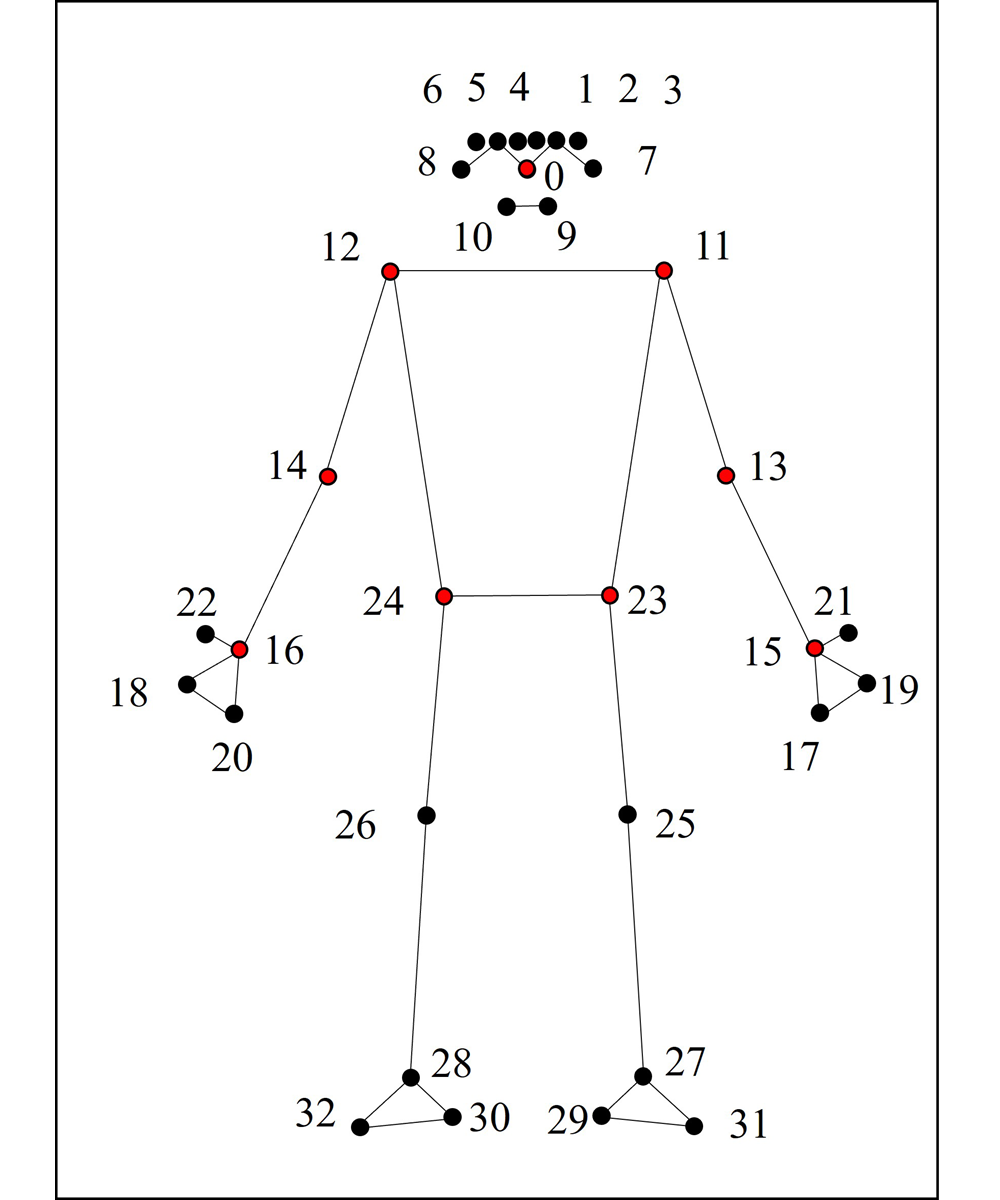

Supplement: Multimedia Appendix 3 [file jmir_v27i1e63308_app3.png]

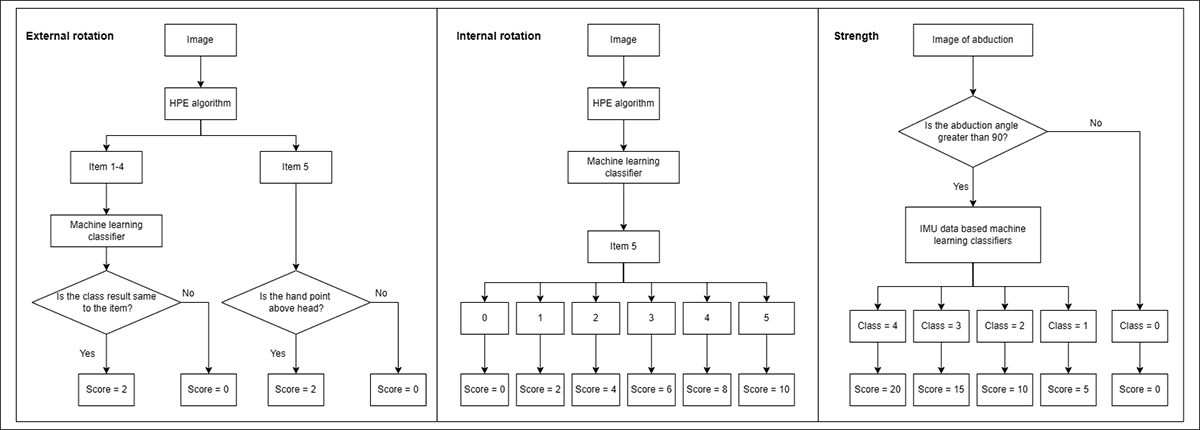

Supplement: Multimedia Appendix 4 [file jmir_v27i1e63308_app4.png]

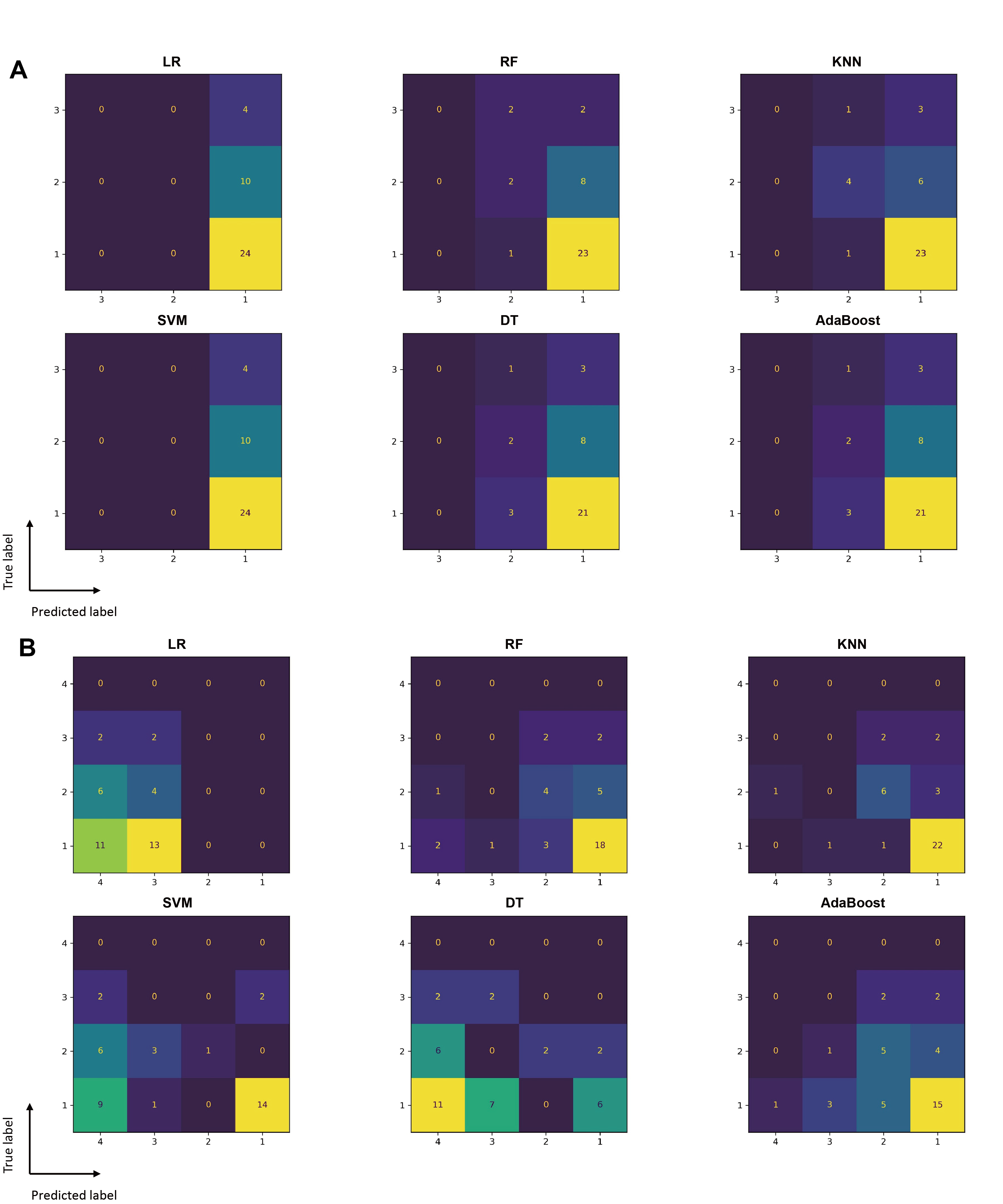

Supplement: Multimedia Appendix 5 [file jmir_v27i1e63308_app5.png]

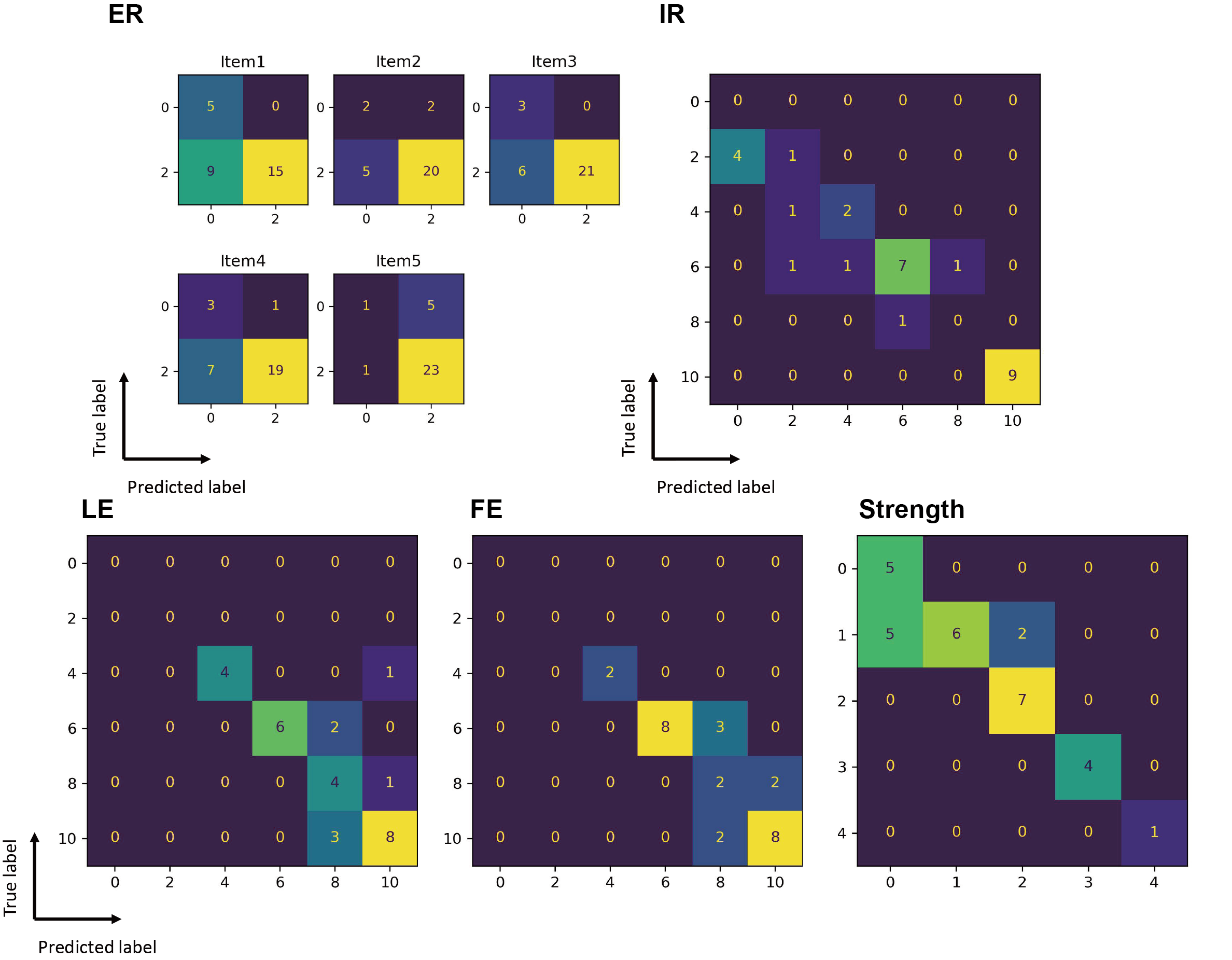

Supplement: Multimedia Appendix 6 [file jmir_v27i1e63308_app6.png]
