# Supplementary material for: The Mobile Constant, a Self-Reported Method for Shoulder Function Evaluation: Development and Validation Study
Source: J Med Internet Res. 2025 Sep 3;27:e63308. doi: 10.2196/63308 (PMC12444215; doi:10.2196/63308)
Supplement: Multimedia Appendix 7 [file jmir_v27i1e63308_app7.docx]

| Parameter name | Calculation | Parameter name | Calculation |
| --- | --- | --- | --- |
| Energy | $X=\sum_{i=1}^{N} x_{i}$ | Root mean square | $X_{rms}=\sqrt{\frac{1}{N}\sum_{i=1}^{N} {x_{i}}^{2}}$ |
| Minimum | $X_{min}=\min\left( x(i) \right)$ | Skewness | $\alpha=\frac{1}{N}\sum_{i=1}^{N} {x_{i}}^{3}$ |
| Maximum | $X_{max}=\max\left( x(i) \right)$ | Kurtosis | $\beta=\frac{1}{N}\sum_{i=1}^{N} {x_{i}}^{4}$ |
| Variance | $Dx=\frac{1}{N}\sum_{i=1}^{N} \left( x_{i}-\bar{x} \right)^{2}$ | Waveform Factor | $S_{f}=\frac{X_{rms}}{\bar{x}}$ |
| Standard deviation | $s =\sqrt{Dx}$ | Peak Factor | $C_{f}=\frac{X_{max}}{X_{rms}}$ |
| Range | $R=x_{\max}-x_{\min}$ | Impulse Factor | $I_{f}=\frac{X_{max}}{\bar{x}}$ |
| Average rectified value | $X_{arv}=\frac{1}{N}\sum_{i=1}^{N} \left\vert\left. x_{i} \right\vert\right.$ | Clearance factor | ${CL}_{f}=\frac{X_{max}}{({\frac{1}{N}\sum_{i=1}^{N} \sqrt{x_{i}})}^{2}}$ |

sTable 1. The calculation of kinemetric parameters
