# Supplementary material for: The Mobile Constant, a Self-Reported Method for Shoulder Function Evaluation: Development and Validation Study
Source: J Med Internet Res. 2025 Sep 3;27:e63308. doi: 10.2196/63308 (PMC12444215; doi:10.2196/63308)
Supplement: Multimedia Appendix 8 [file jmir_v27i1e63308_app8.docx]

sTable 2. Comparison of the accuracy between classifiers with or without oversampling method

| Model name | With oversample | | Without oversample | |
| --- | --- | --- | --- | --- |
|  | Train | Test | Train | Test |
| LR | 33.3 | 6.3 | 52.7 | 63.2 |
| RF | 99.7 | 55.6 | 56.8 | 63.5 |
| KNN | 100.0 | 61.5 | 62.8 | 63.5 |
| SVC | 41.3 | 39.1 | 52.7 | 63.2 |
| DT | 46.9 | 31.3 | 54.2 | 60.2 |
| AdaBoost | 56.3 | 43.8 | 52.9 | 59.9 |

LR: Logistic Regression; RF: Random Forest; KNN: K-Nearest Neighbor; SVM: Support Vector Machine; DT: Decision Tree;
